# Readiness of district and regional hospitals in Burkina Faso to provide caesarean section and blood transfusion services: a cross-sectional study

**DOI:** 10.1186/1471-2393-14-158

**Published:** 2014-05-02

**Authors:** Georges Dayitaba Compaoré, Issiaka Sombié, Rasmané Ganaba, Sennen Hounton, Nicolas Meda, Vincent De Brouwere, Matthias Borchert

**Affiliations:** 1 Centre Muraz, Bobo-Dioulasso, Burkina Faso, PO Box 390, Bobo-Dioulasso, Burkina Faso; 2 AFRICSanté, Bobo-Dioulasso, Burkina Faso; 3 Technical Division, United Nations Population Fund, New-York, NY, USA; 4 Institute of Tropical Medicine, Antwerp, Belgium; 5 Faculty of Epidemiology and Population Health, London School of Hygiene & Tropical Medicine, London, UK; 6 Institute of Tropical Medicine and International Health, Charité – Universitätsmedizin Berlin, Berlin, Germany

**Keywords:** Readiness, Hospitals, Caesarean section, Blood transfusion, Burkina Faso

## Abstract

**Background:**

Health centres and hospitals play a crucial role in reducing maternal mortality and morbidity by offering respectively Basic Emergency Obstetric and Newborn Care (BEmONC) and Comprehensive Emergency Obstetric and Newborn Care (CEmONC). The readiness of hospitals to provide CEmONC depends on the availability of qualified human resources, infrastructure like surgical theatres, and supplies like drugs and blood for transfusion. We assessed the readiness of district and regional hospitals in Burkina Faso to provide two key CEmONC functions, namely caesarean section and blood transfusion. As countries conduct EmONC needs assessments it is critical to provide national and subnational data, e.g. on the distribution of EmONC facilities as well as on facilities lacking the selected signal functions, to support the planning process for upgrading facilities so that they are ready to provide CEmONC.

**Methods:**

In a cross-sectional study we assessed the availability of relevant health workers, obstetric guidelines, caesarean section and blood transfusion services and experience with quality assurance approaches across all forty-three (43) district and nine (9) regional hospitals.

**Results:**

The indicator corresponding to one comprehensive emergency care unit for 500,000 inhabitants was not achieved in Burkina Faso. Physicians with surgical skills, surgical assistants and anaesthesiologist assistants are sufficiently available in only 51.2%, 88.3% and 72.0% of district hospitals, respectively. Two thirds of regional and 20.9% of district hospitals had blood banks. Most district hospitals as opposed to only one third of regional hospitals had experience in maternal death reviews.

**Conclusions:**

Our findings suggest that only 27.8% of hospitals in Burkina Faso at the time of the study could continuously offer caesarean sections and blood transfusion services. Four years later, progress has likely been made but many challenges remain to be overcome. Information provided in this study can serve as a baseline for monitoring progress in district and regional hospitals.

## Background

Although the latest report of the Maternal Mortality Estimation Interagency Group showed a decrease of maternal mortality ratios, the situation in sub-Saharan Africa remains worrisome. In Burkina Faso (actually one of the poorest countries based on the Human Development Index) the maternal mortality ratio was approximately 300 deaths per 100,000 live births in 2010, resulting in 2,100 maternal deaths per year on average [1]. In Burkina Faso like in other sub-Saharan countries haemorrhage is the major cause of maternal deaths, with a prominent role of post-partum haemorrhage [2].

Improving access to skilled attendance at delivery and Emergency Obstetric and Newborn Care (EmONC) are key strategies to reduce maternal mortality and morbidity [3,4]. Health centres and hospitals play a crucial role in reducing maternal mortality and morbidity by providing respectively Basic Emergency Obstetric and Newborn Care (BEmONC) and Comprehensive Emergency Obstetric and Newborn Care (CEmONC) [5-7]. The readiness of hospitals to provide CEmONC depends on the availability of qualified human resources, infrastructure like surgical theatres, and supplies like drugs and blood for transfusion [8]. Recent studies carried out in Africa and Asia pointed out the limited availability of caesarean section and blood transfusion services in many hospitals [9-11]. In order to reduce maternal mortality, the government of Burkina Faso has launched several strategies since 1990 to improve access to skilled attendance and EmONC; such strategies include the development of infrastructure (health centres, hospitals with surgical theatres), the recruitment and training of health workers, the reduction of user fees for maternity care through government subsidies and the introduction of generic drugs. To supplement the low number of gynaecologists, the Burkina government has been promoting a new type of human resource for performing C-section in district hospitals since 1992 by offering a six-month training in Basic Emergency Surgery (caesarean section, laparotomy with uterus repair, incarcerated hernias etc.) to general medical practitioners [12]. According to national standards, each district hospital should have at least two such General Practitioners trained in Basic Emergency Surgery (GP-BES). To support gynaecologists, surgeons and GP-BES, at least two surgical assistants (nurses with two years training in surgery) and two anaesthesiologist assistants (nurses with two years training in anaesthesia) should be present in each district hospital as well.

Despite the implementation of these strategies several studies have documented a low number and poor distribution of qualified workers, a low quality of care, and resulting shortcomings in the functionality of the health system of Burkina Faso [13,14]. There is hardly any routine information about the readiness of Burkina’s hospitals to provide EmONC. Readiness is defined as “achieving and maintaining a state of preparedness in the facility to provide quality emergency obstetric care. This includes sufficient numbers of staff members available with requisite skills and a willingness to respond to clients 24 hours a day, 7 days a week, available and functional equipment and supplies, and adequate infrastructure” [15].

In this article we report on the readiness of district and regional hospitals of Burkina Faso to provide two key functions of CEmONC in 2007. Since then, Burkina Faso has conducted a national EmONC needs assessment in 2011 [16], so that we could, within limitations, assess changes over time for some indicators. Our study was conducted to prepare the cluster-randomised trial “Effectiveness of facility-based audits to improve the responsiveness of West African district hospitals to obstetric emergencies (AUDOBEM, registered as ISRCTN67206260)” conducted in Benin, Burkina Faso and Niger, on which we shall report elsewhere.

## Methods

In Burkina Faso, the health system consists of various levels, each of which is designed to provide a range of services (in brackets the type of health facility and the kind of EmONC it normally provides): the peripheral level (health centres; BEmONC), the lower intermediate level (district hospitals; CEmONC), the upper intermediate level (regional hospitals; CEmONC and occasionally specialist obstetric care) and the central level (national teaching hospitals; CEmONC and specialist obstetric care). At the time of the study - in 2007 - there was either a national teaching hospital (n = 2) or a regional hospital (n = 9) in 11 out of 13 administrative regions, and a regional hospital (n = 9), a district hospital with surgery unit (n = 42) or a district hospital without surgery unit (n = 1) in 52 out of 53 health districts. The district of Tô had only one health centre and was therefore excluded from this survey. Regional hospitals operated both as district hospitals in the districts where they were located and as referral hospitals for the other districts of their region. All hospitals with the exception of two confessional district hospitals were governmental.

Between March and April 2007, we conducted a cross-sectional study in all district and regional hospitals in Burkina Faso. The two national teaching hospitals located in Ouagadougou and Bobo-Dioulasso were taken into account only when estimating the continuous availability of CEmONC. We considered districts located in the two main cities - Ouagadougou and Bobo-Dioulasso - as urban districts, and all other districts as rural ones.

For the purpose of this study we defined as the absolute minimum number of required staff in CEmONC facilities to be three midwives (one each for morning, afternoon and night duty) to guarantee the presence of a skilled birth attendant twenty four hours a day, seven days a week in the maternities, and two surgical assistants and two anaesthesiologist assistants for providing 24/7 assistance to physicians with surgical skills.

We assessed both the availability of health workers of relevant categories (physicians including gynaecologists, surgeons, general practitioners trained in emergency surgery and general practitioners without training in basic emergency surgery, surgical assistants, anaesthesiologist assistants, midwives and auxiliary midwives) and the availability of obstetric guidelines.

We used the availability and frequency of caesarean sections and the availability of blood transfusion services to judge the readiness of hospitals to provide CEmONC, since these are the two key functions distinguishing CEmONC from BEmONC, i.e. the two EmONC functions expected to be provided by district hospitals and above only, not by health centres. We qualified the availability of C-section as intermittent when this service was available sometimes but not 24/7 at the time of the survey. We considered blood transfusion services to be continuously available when the hospital had a blood bank, and intermittently available when blood was provided by family members or through a list of blood donors.

We defined a hospital as ready if both two key functions were continuously available, as not ready if neither key function was available at all, and as partially ready when either key function was intermittently available.

The study was conducted by an epidemiologist visiting all district and regional hospitals. Interviews with hospital managers and registers were sources of information. Data were entered with EpiInfo™ (Center for Disease Control and Prevention, Atlanta, GA) and analyzed with SPSS 12.0 (IBM Corporation, Armonk, NY). We compared proportions of categorical data and medians of continuous variables using the chi-square test. Differences were considered as statistically significant when P < 0.05.

The study was approved by the Institutional Review Board of Centre Muraz. Verbal informed consent was obtained from hospital staff members who participated in the survey.

## Results

### Availability of human resources for CEmONC

The distribution of health workers in district and regional hospitals is shown in Table 1. In the 43 district hospitals, we identified a total of 105 physicians including ten gynaecologists, three surgeons, 56 GP-BES and 36 general practitioners without surgical skills. All ten gynaecologists worked in district hospitals in Ouagadougou, the capital city. One of the three surgeons was located in a district hospital of the capital city while the other two worked in two rural district hospitals. The 56 GP-BES were located in 37 district hospitals. 22 district hospitals (51.2%) had at least two physicians with surgical skills (gynaecologists, surgeons, GP-BES); 18 hospitals (41.8%) had at least two GP-BES.

**Table 1:** Availability of human resources for CEmONC in district and regional hospitals, Burkina Faso, 2007

	**Total number**	**Mean number per hospital**	**% hospitals with a specific number of health workers**						
			**0**	**1**	**2**	**3**	**4**	**5**	**> 5**
**District hospitals (n = 43)**									
Physicians, total	105	2.4	0.0	4.6	65.1	18.6	7.0	2.3	2.3
Physicians with surgical skills	69	1.6	4.6	44.2	41.9	7.0	0.0	2.3	0.0
Gynaecologists	10	0.2	90.7	2.3	4.6	0.0	0.0	2.3	0.0
Surgeons	3	0.1	93.0	7.0	0.0	0.0	0.0	0.0	0.0
GPs trained in basic emergency surgery*	56	1.3	14.0	44.2	39.5	2.3	0.0	0.0	0.0
GPs without surgical skills*	36	0.8	37.2	41.9	20.9	0.0	0.0	0.0	0.0
Surgical assistants	106	2.5	4.6	7.0	48.8	25.6	9.3	2.3	2.3
Anaesthesiologist assistants	93	2.2	2.3	25.6	55.8	7.0	4.6	0.0	4.6
Midwives	169	3.9	0.0	0.0	30.2	34.9	9.3	11.6	13.8
Auxiliary midwives	222	5.2	7.0	2.3	11.6	7.0	14.0	20.9	37.2
**Regional hospitals (n = 9)**									
Physicians, total	23	2.5	0.0	33.3	0.0	55.6	0.0	11.1	0.0
Physicians with surgical skills	20	2.2	0.0	44.4	11.1	33.3	0.0	11.1	0.0
Gynaecologists	14	1.5	0.0	44.4	55.6	0.0	0.0	0.0	0.0
Surgeons	4	0.4	66.7	22.2	11.1	0.0	0.0	0.0	0.0
GPs trained in basic emergency surgery*	2	0.2	77.8	22.2	0.0	0.0	0.0	0.0	0.0
GPs without surgical skills*	3	0.3	77.8	11.1	11.1	0.0	0.0	0.0	0.0
Surgical assistants	58	6.4	0.0	0.0	0.0	0.0	0.0	33.3	66.7
Anaesthesiologist assistants	43	4.8	0.0	0.0	11.1	11.1	0.0	44.4	33.3
Midwives	84	9.3	0.0	0.0	0.0	0.0	0.0	0.0	100.0
Auxiliary midwives	18	2.0	22.2	11.1	44.4	0.0	11.1	11.1	0.0

*GPs: General practitioners.

The required minimum number of three midwives was met in 30 district hospitals (69.4%), of two surgical assistants in 38 district hospitals (88.3%), and of two anaesthesiologist assistants in 31 district hospitals (72.0%).

The distribution of health workers in urban and rural district hospitals is shown in Table 2. The median numbers of midwives were 10 (range 6–16) in urban district hospitals, against 3 (2–12) in rural district hospitals (p = 0.039, Chi^2^-test for comparison of medians, with Pearson continuity correction). The median number of anaesthesiologist assistants was respectively 4 (3–9) and 2 (0–6) for urban hospitals and rural district hospitals (p = 0.001).

**Table 2:** Distribution of health workers in urban district hospitals and rural regional and district hospitals, Burkina Faso, 2007

**Health workers category**	**Urban district hospitals N = 5**			**Rural regional and district hospitals N = 47**			**P (for medians)**
	**Median**	**Minimum**	**Maximum**	**Median**	**Minimum**	**Maximum**	
Physicians	4	2	6	2	1	5	0.511
Midwives	10	6	16	3	2	12	0.039
Surgical assistants	4	3	7	2	0	10	0.060
Anaesthesiologist assistants	4	3	9	2	0	6	0.001
Auxiliary midwives	10	6	13	4	0	11	0.060

### Availability of guidelines and experience with quality assurance approaches

The availability of relevant guidelines (for reproductive health, emergency obstetric and newborn care, management of complicated pregnancies and deliveries) in the hospitals varied from 11% to 64%. Reviews of maternal deaths or complications were conducted in more than 80% of the district hospitals against only in one third of the regional hospitals (Table 3).

**Table 3:** Availability of safe motherhood guidelines, and experience with quality improvement approaches in district and regional hospitals, Burkina Faso, 2007

	**District hospitals N = 43%**	**Regional hospitals N = 9%**
**Availability of guidelines**		
% with national reproductive health guidelines	64.3	33.3
% with emergency obstetric care guidelines	19.1	44.4
% with guidelines on complicated pregnancy and deliveries	11.9	11.1
% with at least one team member trained to use any of the three above	19.1	22.2
**Quality assurance approaches**		
% with experience in maternal death review	81.0	33.3
% with experience in review of maternal complications	4.8	0.0
% with at least one team member trained in any of the two above	61.9	44.4

### Frequency of deliveries

Table 4 describes the deliveries recorded in the district and regional hospitals of Burkina Faso in 2006. The proportions of C-sections performed in regional versus district hospitals that were ready were almost identical (respectively 21.2% versus. 21.5%). When hospitals were partially ready, regional hospitals had significantly higher proportions of C-sections than district hospitals (17.0% versus. 7.4%, p < 0.001). Comparing hospitals by degree of readiness, partial readiness reduced the proportion of C-sections in district hospitals by 14.1%, but by 4.2% only in regional hospitals.

**Table 4:** Frequency of deliveries in district and regional hospitals in Burkina Faso, 2006

	**District hospitals N = 42**^**a**^			**Regional hospitals N = 9**		
	**Ready N = 7**	**Partially ready N = 35**	**All**	**Ready N = 6**	**Partially ready N = 3**	**All**
Deliveries	5 187	20 717	25 904	5 841	3 147	8 988
C-sections	1 118	1 543	2 661	1 241	534	1 775
% of C-sections	21.5	7.4	10.3	21.2	17.0	19.7

^a^:only takes into consideration hospitals that were ready or partially ready.

### Availability of key CEmONC functions

Table 5 shows the availability of C-section and blood transfusion services in district and regional hospitals. Two thirds of regional and 20.9% of district hospitals had blood banks. In the other regional hospitals and in most district hospitals it was possible to perform a blood transfusion by calling on volunteers or family members. In six district hospitals, no blood transfusion was possible.

**Table 5:** Availability of key CEmONC functions in district and regional hospitals in Burkina Faso, 2007

	**District hospitals N = 43 (%)**	**Regional hospitals N = 9 (%)**
**C-section**		
Never available^a^	4 (9.3)	0 (0.0)
Available intermittently	16 (37.2)	0 (0.0)
Available continuously	23 (53.5)	9 (100)
**Blood transfusion services**		
Never available	6 (13.9)	0 (0.0)
Available intermittently^b^	28 (65.1)	3 (33.3)
Available continuously^c^	9 (20.9)	6 (66.7)
**Readiness of hospitals to provide CEmOC**		
Not ready	1 (2.3)	0 (0.0)
Partially ready	35 (81.4)	3 (33.3)
Ready	7 (16.3)	6 (66.7)

^a^:C-section neither at the time of the survey nor during the year 2006.

^b^:through family members or list of blood donors for district hospitals, only through list of blood donors for regional hospital.

^c^:through blood bank.

**Figure 1:**
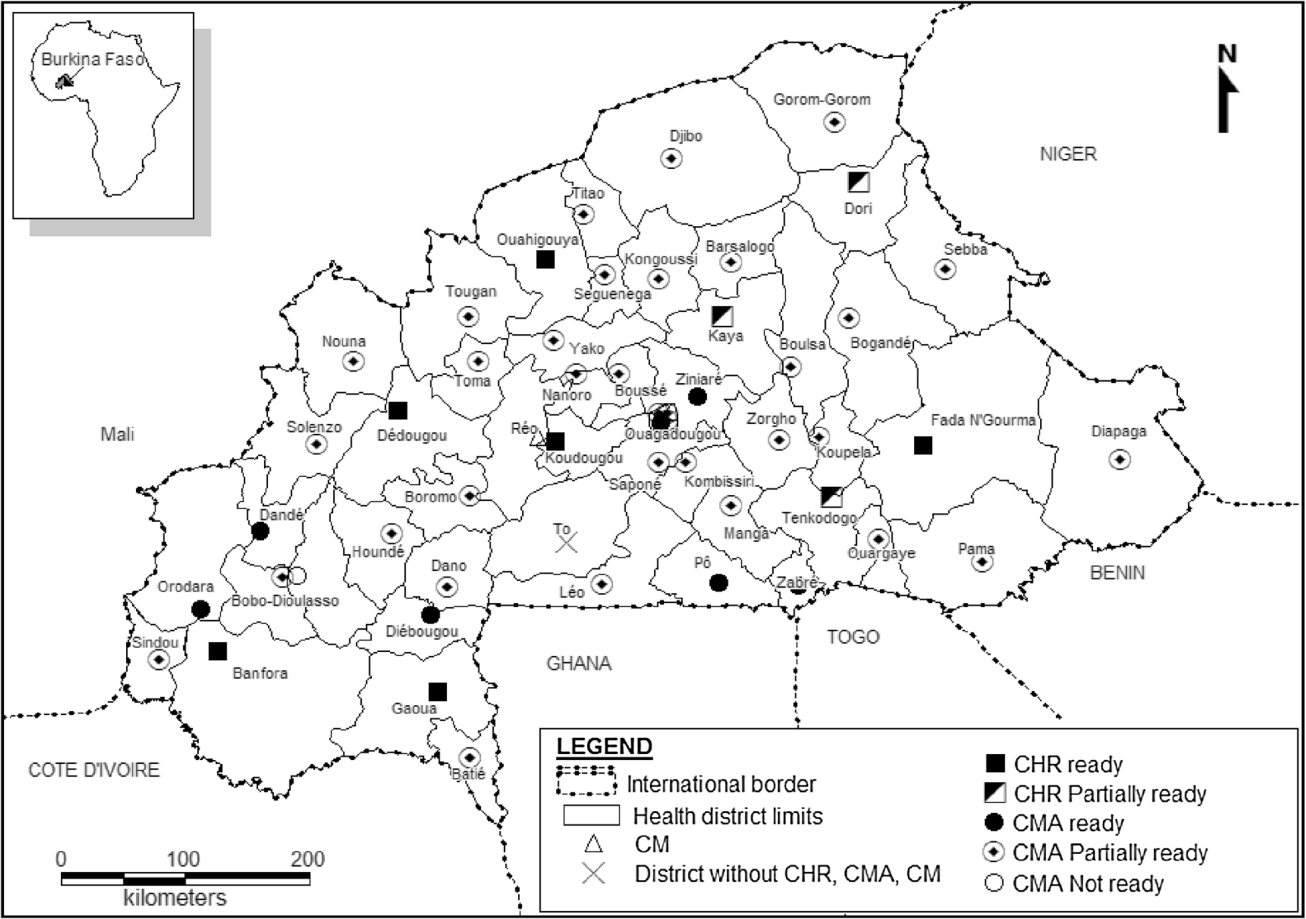
**Readiness of regional and district hospitals based on the availability of caesarean and blood transfusion services, Burkina Faso, 2007.** CHR: Regional hospital, CMA: District hospital with surgery unit, CM: District hospital without surgery unit.

Figure 1 shows a relatively good geographical distribution of both regional and district hospitals within the country, very few of which are ready to provide CEmONC. According to the inter-agency handbook on monitoring EmONC [17] there should be one CEmONC unit for 500,000 inhabitants, in addition to four BEmONC units. With an estimated population of 13,000,000 inhabitants in 2007, this translates into a minimum of 26 CEmONC units for Burkina Faso. With 15/54 hospitals (27.8%; 2/2 national teaching hospitals, 6/9 regional and 7/43 district hospitals) offering caesarean section and blood transfusion services 24/7, this norm was not achieved.

## Discussion

We found a lack of qualified human resources, coupled with higher availability of physicians and midwives in regional hospitals than in district hospitals, and higher availability in urban than in rural district hospitals. Clinical guidelines were not always available, and insufficient numbers of staff were trained in their use. There were gaps in the provision of C-sections and blood transfusions in some district hospitals.

**Figure 2:**
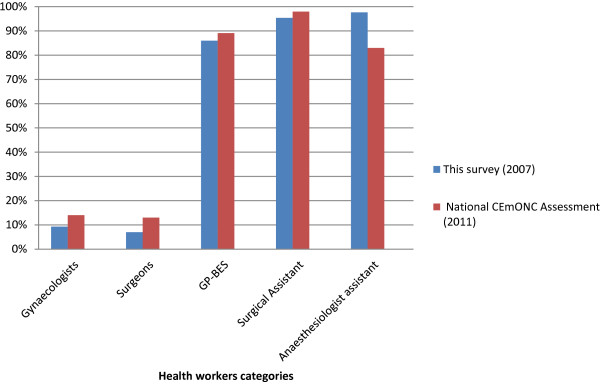
Percentage of district hospitals with at least one health worker of various categories in this survey (2007) and the National CEmONC Assessment (2011).

The lack of qualified human resources in district hospitals in sub-Saharan Africa is not new and has been pointed out by several studies [9,13,14]. To address the shortage of obstetricians in district hospitals of sub-Saharan Africa, some countries like Ethiopia, Malawi, Mozambique and Tanzania use non-physician clinicians to perform emergency surgery including C-section [18-21]. In 1992 Burkina Faso introduced the training of GP-BES (including C-section) to ensure functionality of district hospitals as the first referral level of its health system [12]. Although this strategy has been in place in Burkina Faso for more than fifteen years, 48.8% of all district hospitals did not even have two physicians with surgical skills, meaning that caesarean sections could not be provided on a continuous basis. We found that 30.2% of district hospitals had less than three midwives, so that continuous availability of skilled birth attendance was not assured either. The scarcity of qualified staff should be considered as even more pronounced than suggested by our figures: in reality more than two physicians with surgical skills, two surgical assistants, two anaesthesiologist assistants and three midwives are necessary to ensure continuous availability of CEmONC, since the need for rest after night duty and for replacing staff on annual or sick leave need to be taken into account. The scarcity of skilled human resources can explain the low availability and quality of EmONC in the district hospitals identified in several studies in Burkina Faso [12,16,22]. Significant progress was achieved in the four years following our survey with respect to human resources. A comparison of our results from 2007 with those of the national CEmONC assessment in 2011 [16] shows an increase of the proportions of district hospitals with at least one gynaecologist, surgeon, GP-BES and surgical assistant, but not with at least one anaesthesiologist assistant (Figure 2). The efforts of the government to improve the availability of qualified human resources in district hospitals should continue in order to achieve the maternal mortality reduction required by Millennium Development Goal (MDG) 5.

We found that guidelines on obstetric and newborn care were less readily available than guidelines on reproductive health in general, and in 80% of the hospitals no team member had been trained in the use of these guidelines. Not only should the availability of obstetric and newborn clinical guidelines be improved, but a critical mass of health workers should be trained for a continuous improvement of the quality of care in spite of the usual staff turnover. Unless qualified human resources are made available in sufficient numbers to carry out emergency surgery, emergency referrals may be the only option for many life-threatening complications. Having obstetric guidelines and health workers trained to use them can help to identify such complications at an early stage, and can prompt a timely referral.

Experience with maternal death reviews was widespread in district hospitals, but limited in regional hospitals. Maternal morbidity reviews ranged from rarely conducted in district hospitals to not conducted in regional hospitals. Respectively 55.6% and 38.1% of the regional and district hospitals had no worker trained in conducting such reviews, which underlines the need for more in-service training as well as the integration of review practices into pre-service training.

As expected, regional hospitals had higher proportions of C-sections than district hospitals, regardless of their readiness to provide CEmONC. This result is consistent with the role of regional hospitals to provide secondary care and an opportunity for district hospitals to refer patients. The difference in proportions of C-sections between regional hospitals and district hospitals that are ready suggests that most district hospitals did not entirely fulfil their role as CEmONC-providing health facilities. Particular attention should thus be paid to the upgrading of district hospitals so they can contribute to strengthening the health system and to reducing maternal mortality.

The limited availability of EmONC in sub-Saharan African has been pointed out before. The number of hospitals offering caesarean sections and blood transfusion services 24 hours a day, 7 days a week was only half the required number according to the recommendations by the inter-agency handbook on monitoring EmONC [17]. They are complemented by hospitals offering these interventions intermittently; these hospitals need upgrading for offering caesarean sections and blood transfusion services continuously. A non-negligible proportion of district hospitals of Burkina Faso are unable to perform any caesarean sections (9.3%) or blood transfusions (13.9%). A comparison of our results from 2007 with those of the national CEmONC assessment from 2011 [16] suggests that some progress may have been made in the continuous provision of caesarean sections (53.5% in 2007 vs. 88.4% in 2011) and of blood transfusion services (20.9% vs. 76.7%) in district hospitals. A direct comparison of these results, however, is problematic, since the two studies used different definitions of continuous availability: in 2011 continuous availability of relevant staff alone was sufficient to declare that caesarean sections were available continuously, while in 2007 shortage of supplies, electricity, water etc. as well as shortage of staff could have led to caesarean sections to be declared as not continuously available. For blood transfusion, similar limitations for the comparison of results from 2007 versus 2011 apply. Progress may have been more modest than suggested by the figures, but Burkina’s health system has probably moved in the right direction.

Improving the availability of caesarean sections is thus an important challenge for the future. While our survey has not comprehensively assessed all possible reason for the lack of continuous provision, it has revealed that further investments in human resources are warranted. Improving the availability of blood transfusions is another important challenge. The national programme of blood transfusion in collaboration with the Luxemburg government recently trained human resources, and built and equipped blood banks. This programme could potentially improve the availability of blood transfusion in referral hospitals of Burkina Faso. It should closely collaborate with the Safe Motherhood programme of the ministry of health to train maternity health workers on when and how to perform safe blood transfusion.

The results of our survey and those of the EmONC needs assessment [16] four years later suggest that progress has likely been made but many challenges remain to be overcome. They suggest that the government should make it a priority to improve the availability and the quality of EmONC in order to achieve a significant reduction in maternal and newborn mortality in Burkina Faso.

### Strength and limitations

The main strength of this study was its comprehensive coverage of all district and regional hospitals. No selection bias could have occurred, so the results are representative for the country.

This study has four main limitations. Firstly, we focussed only on selected key functions of CEmONC (caesarean section and blood transfusion), and did not include signal functions such as basic neonatal resuscitation which are part of both BEmONC and CEmONC. Secondly, the cross-sectional design of our study prevents us from detecting trends in the capacity of district hospitals to provide C-section and blood transfusion services. Thirdly, for some variables of our study (experience with quality assurance approaches, availability of human resources etc.), we relied on interviews instead of documentary evidence, like health facilities statistics. Lastly, we operationally define the availability of blood transfusions as continuous when provided by a blood bank, which may not always be a valid definition.

## Conclusions

Our findings suggest that only 27.8% of hospitals in Burkina Faso at the time of the study could continuously provide caesarean section and blood transfusion services. Four years later, progress has likely been made, but many challenges remain to be overcome. With these substantial gaps in CEmONC coverage, formidable efforts are necessary to achieve the MDG 5 target of 175 maternal deaths per 100,000 live births in Burkina Faso [23]. Information provided in this study can serve as baseline for monitoring progress in district and regional hospitals.

## Abbreviations

BEmONC: Basic Emergency Obstetric and Newborn Care; CEmONC: Comprehensive Emergency Obstetric and Newborn Care; GP-BES: General Practitioner trained in Basic Emergency Surgery; MDG: Millennium Development Goal.

## Competing interests

The authors declare no competing interest.

## Authors’ contributions

Designing the study: IS, RG, SH, NM, MB. Data collection: RG. Analysing the data: GDC, IS. Interpreting results: GDC, IS, MB. Drafting the manuscript: GDC, IS. Critically reviewing the manuscript: RG, SH, NM, VDB, MB. All authors read and approved the final manuscript.

## Pre-publication history

The pre-publication history for this paper can be accessed here:

http://www.biomedcentral.com/1471-2393/14/158/prepub
